# HAZOP Methodology Based on the Health, Safety, and Environment Engineering

**DOI:** 10.3390/ijerph17093236

**Published:** 2020-05-06

**Authors:** Jae-Young Choi, Sang-Hoon Byeon

**Affiliations:** Department of Health and Safety Convergence Science, Korea University, Anam-ro 145, Seongbuk-gu, Seoul 02841, Korea; jaeyoungchoi@korea.ac.kr

**Keywords:** risk analysis, HAZOP, process plant, HSE engineering, operator safety, chemical engineering, process safety

## Abstract

In existing risk analysis techniques like the hazard and operability study (HAZOP) and the safety integrity level (SIL), design for operator safety is not considered. The health, safety, and environment (HSE) engineering depicts a detailed design directly related to the operator safety. However, the human risk had not been comprehensively analyzed. This paper proposes HSE-HAZOP as a technique for examining the systematic and efficient application of HSE engineering by exploiting the HAZOP systematic risk analysis technique and a quantitative risk derivation method, which is an advantage of the SIL. The analysis consists of four steps: the HSE-HAZOP preparation phase, risk analysis phase, risk assessment phase, and risk reduction phase. One part of a solution styrene butadiene rubber (SSBR) plant was used for a case study. In this case study, the items that handle with heptanoic acid were the study scope. After the risk assessment, we introduced the HSE engineering technique that should be applied for the risk reduction. Since there is no existing risk analysis method for HSE engineering, this proposed HSE-HAZOP is meaningful because it suggests systematic analysis method of the operator safety.

## 1. Introduction

Hazard and operability study (HAZOP) is applied worldwide as one of the process hazard analysis (PHA) techniques for processing plants [1]. HAZOP is implemented on a legal basis for the licensing of processing plants in accordance with the process safety management of the US Occupational Safety and Health Administration (OSHA) and the Seveso provisions of the EU [2]. HAZOP was first proposed by Imperial Chemical Industries in 1960, was developed by the Chemical Industries Association in 1970, and eventually became a global standard of IEC 61882 [3]. 

However, HAZOP is centered on the piping and instrument diagram (P & ID) of process engineers [4] and neglects health, safety, and environment (HSE) engineering, which depicts a detailed design directly related to the operator safety, as it considers the stability of the process for the smooth production of chemical products. The cause of Seveso incident that happened on 10 July 1976 was not a lack of knowledge, but was caused by the lack of a tool to precisely analyze the knowledge. Therefore, the research on the development and improvement of tools for safety evaluation has been conducted in the past and present, and there have been AcciMap approach, the Energy Barrier Model, and the System-Theoretic Accident Model and Processes [5]. In addition, a study was recently conducted on the improvement of HAZID, one of the PHA techniques [6]. This type of research is a field that has been conducted steadily since the loss prevention department was established by an insurance company in 1960. However, the research on PHA techniques was just focused on the process issues, and there were no studies that combined it with HSE engineering. Therefore, this study aims to improve HAZOP, one of the representative PHA techniques, by combining HSE engineering.

As shown in Table 1, the process plant is designed according to seven major disciplines [7]. In HAZOP, which is central to process engineering, it is difficult to consider HSE engineering factors.

In HSE engineering, detailed design is performed, starting with the definition of a toxic fluid [8]. Typical design achievements are presented in Table 2.

Because the P & ID produced by process engineers [9] does not provide detailed information regarding toxic fluids, HAZOP cannot consider HSE engineering using the HAZOP review of the P & ID as the basis. In contrast to the P & ID, the process flow diagram (PFD) and the heat and material balance (HMB), which are other design documents of a process plant, provide detailed information regarding the composition of materials in each process flow. Using the PFD and HMB, the process stream that deals with toxic substances above a certain standard value can be identified, and it is possible to depict which items deal with specific toxic substances.

Herein, we propose a method called HSE-HAZOP, which uses the PFD and HMB as bases, in contrast to the HAZOP review of the P & ID. HSE-HAZOP is based on systematic risk assessment, which is a merit of HAZOP, and it is suggested that successful HSE engineering can be achieved through risk quantification and risk reduction using the safety integrity level (SIL).

## 2. Existing PHA Method

The HAZOP and SIL are based on global standards (IEC 61882 for HAZOP and IEC 61511 for SIL). In this section, we introduce the existing HAZOP and SIL, their limitations, and the improvements that are possible via their combination with HSE engineering.

### 2.1. HAZOP

HAZOP starts by selecting a node that is an isolation point constituting the flow of the P&ID valve or equipment from the process viewpoint. According to the process knowledge of the chairperson, which is the key role of HAZOP, this node is divided into manageable segments of the system, which require independent reviews of the process deviations. Therefore, the process engineer and the chairperson are central to the process, and reviews are inevitable. Additionally, HAZOP nodes are not based on HSE engineering factors, because they reflect process issues rather than operator stability.

**Figure 1:**
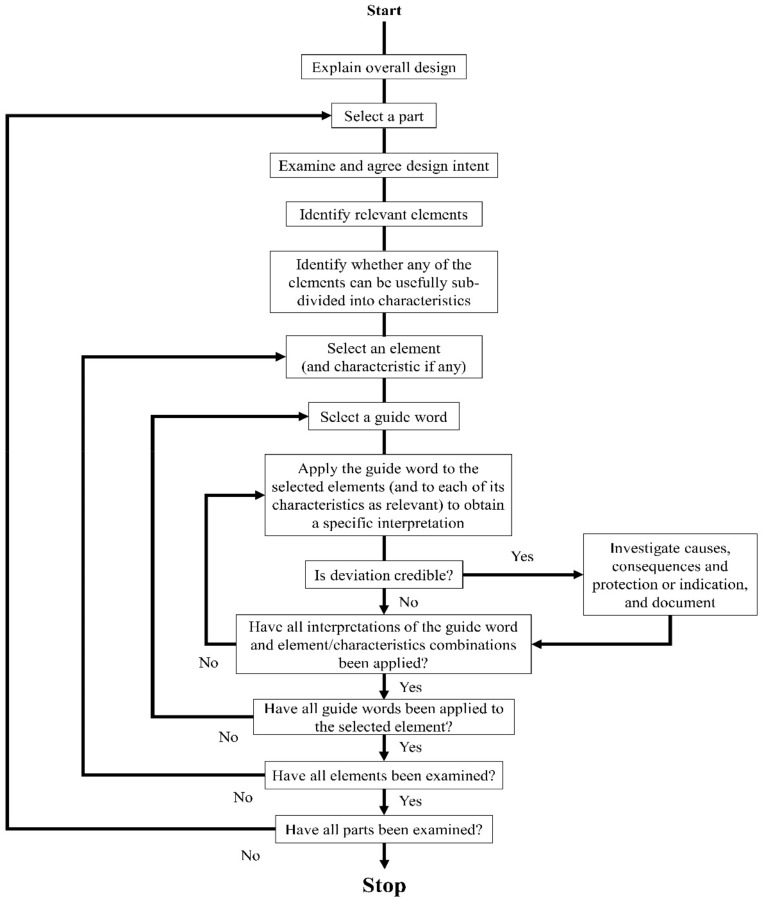
Flowchart of the HAZOP examination procedure.

In HAZOP, engineers from various disciplines gather in a workshop and review (via brainstorming) the issues related to the corresponding node section. The detailed procedure is shown in Figure 1 [10].

The proposed HSE-HAZOP method employs the HAZOP workshop method, in which various discipline engineers gather and systematically review the process issues, using it as a methodology for HSE engineering applications.

### 2.2. SIL

**Figure 2:**
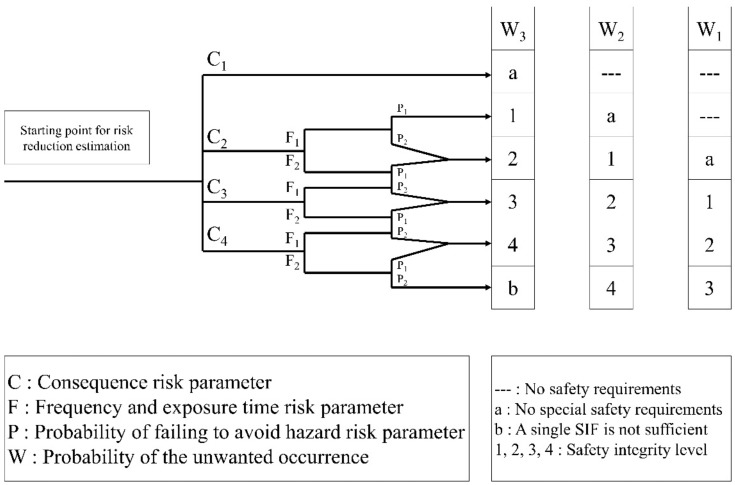
SIL risk graph for personnel safety.

The SIL is a type of PHA that indicates whether the safety instrumented system (SIS) is sufficient to ensure safety through quantitative review of the safety instrumented function (SIF). The risk in the SIL is divided into human injury, property damage, and environmental damage. However, the SIL is ultimately aimed at applying the rating of the facility based on the failure rate of the SIS, for which the control and instrumentation engineer is responsible. Therefore, there is no direct connection with the application of HSE engineering. In the case of the SIL, a systematic methodology quantitatively classifies the risks. Here, the analytical method for human safety is shown in Figure 2 [11]. In this study, we conduct a quantitative evaluation of risk scenarios derived from HSE-HAZOP by modifying and evaluating the review of human safety related to HSE engineering. Additionally, HSE engineering is regarded as independent protection layer and is used for risk reduction.

## 3. HSE Engineering

HSE engineering is derived from the growing complexity of processing plants, as process engineers become aware of the need to introduce a safety perspective into their designs [12]. Since its introduction in 1974, HSE engineering has evolved, with many advancements [13]. Currently, HSE engineering is an independent part of the process design, and it has developed into a method for predicting risk by using design and scientific simulation tools for assessment of human damage factors in the process plant [14]. The HSE engineer is different from the process engineer in that the HSE engineer regards the design target as a human factor related to the process plant [15]. In this section, the representative elements of HSE engineering are divided into four parts.

### 3.1. Safety Items: Design for Equipment That Can Physically Protect Operator at Risk

A safety item is equipment that is primarily used to protect an operator working in a process area exposed to a risk situation. There are three typical configuration items.

1. SSEW
2. PPE
3. SCBA

The SSEW is a facility for flushing water in emergencies, if the operator is directly exposed to toxic fluid through skin or eye contact. The SSEW is based on the international standard ANSI Z358.1. The PPE and SCBA reduce the frequency of exposure to toxic fluid. The PPE is designed with reference to NFPA 1500 and NFPA 1971, and the SCBA is designed with reference to NFPA 1981.

### 3.2. Support Safety Management System: Safety Improvement Through Training of Operators

One measure to reduce the damage in safety management, when the operator working in the process plant encounters a risk situation, is safety improvement via training. This differs from the safety items that reduce the primary risk. There are three typical configuration items.

1. Operating manual revised according to HSE
2. EERA
3. Workplace monitoring program

The operating manual is created by the process engineer; however, it may be requested for additional HSE risk situations that have not been recognized, allowing the operator to act appropriately in response to the risk situation. The EERA collectively refers to measures to minimize personal injury in the risk situation faced by the operator, such as an emergency response plan or an escape route design. The EERA is based on NFPA 101. The workplace monitoring program can contribute to safety management, allowing safety to be maintained by performing an operational exposure assessment of the operator working in the process plant. The workplace monitoring program may consider work measurement cycles for certain substances, in accordance with OSHA standards.

### 3.3. Safety Facility: Design of Equipment That Can Improve Work Safety of Operators

A safety facility employs techniques that introduce design elements into a process plant to avoid risk situations. Here, the risk is prevented as a design element of the process plant for a risk situation that cannot be covered by a safety item or a support safety management system. There are four typical configuration items.

1. Safety fence and sign
2. Dike
3. Toxic-gas detector
4. Damper inside the building

The safety fence and sign are installed for restricting operator access to areas where risk is expected. The dike reduces the evacuation time of the operator when internal material leaks from the equipment, such as a storage tank. Typically, its design is performed according to NFPA 30. The toxic-gas detector informs the operator in the central control building when toxic gas leaks from a facility inside the process plant, so that the operator can shut down a specific process. Toxic-gas detectors are designed with reference to NFPA 72. The damper inside the building prevents the exposure of workers in the building to toxic fluid from the outside of the building.

### 3.4. Simulated Safety Design: Design to Ensure Safety of Operators Through Simulation

Recently, HSE engineering was utilized for risk analysis using a simulation tool [14]. The influence of the toxic-gas dispersion inside and outside the building can be examined according to the layout of the chemical plant. There are two representative examples.

1. QRA: toxic-gas dispersion study
2. OCA

In both QRA and OCA, the major accident hazard is selected, and the influence of toxic gas dispersed from the corresponding source is confirmed through simulation. The diffusion endpoint of the toxic gas is generally applied to the emergency response planning guideline value of the gas. In the case of the major accident hazard selection, a third party or the engineer of the process plant owner is selected to perform QRA and OCA. In particular, OCA is mandated by the U.S. Environmental Protection Agency [16]. In contrast to QRA, in OCA, it is important to examine whether the diffusion of toxic gas in the process plant affects the residents near the process plant [17].

## 4. HSE-HAZOP Methodology

The proposed HSE-HAZOP method is based on the HAZOP systematic analysis method and the SIL quantitative risk calculation method for issues that require HSE engineering. It is divided into the following four stages.

### 4.1. HSE-HAZOP Preparation Phase

#### 4.1.1. Definition of Toxic Service

HSE engineering starts with selecting equipment to deal with toxic services. A toxic service in a process plant is referred to as a lethal service. According to the ASME Code, device machinery dealing with a lethal service should comply with a specific design [18]. As such, a “lethal service” simply refers to a substance for which a design action beyond that required for a toxic service is necessary. However, there is no clear international code or standard providing guidelines for lethal services [19]. Therefore, the owner of a process plant usually requires the lethal services to be separately defined and considered in the design. For Saudi Aramco, lethal services are selected for installations that handle H_2_S in concentrations exceeding 20 vol% [20]. Shell has a comprehensive reference providing standards for not only H_2_S but also LD50 and LC50, and the facilities covered by the relevant substances as lethal service. Details are presented in Table 3 [21].

This standard was derived via consultation with the 28th OECD Chemical Committee in November 1998 and the chemical industry [22]. However, in the present study, we apply the more conservative UN Globally Harmonized System (GHS) and International Labour Organization (ILO) standards. In these standards, toxic services are divided into five categories, which provide ranges of LD50 and LC50 for each section. Details are presented in Table 4 [23].

Table 5 presents the effect of each category with regard to toxicity.

As shown in Table 5, substances in category 3 can have a fatal impact on the human body. In Korea, lethal service is classified according to category 3 of Table 5, and measures to supplement the design of a process plant that deals with this type of service are necessary. Therefore, in this study, HSE engineering is applied to the items that deal with substances corresponding to category 3 of toxic services, as specified by the UN GHS and ILO standards.

#### 4.1.2. Selection of Lethal Service Through HMB

In the PFD, the process flow between the pieces of equipment is indicated by numbers. The composition of the material corresponding to each of these numbers is mentioned in the HMB. By separating the HMB stream that deals with the material selected by the lethal service, one can see which stream is handling the lethal service.

#### 4.1.3. Markup of Lethal Stream in PFD

When the number of the stream handling the lethal service is determined, the corresponding stream is marked up in the PFD. There are several process streams in the PFD. The markup can be used to visually check which equipment is handling the lethal service.

#### 4.1.4. Listing of Lethal Service Items

Items are listed through the markup stream. In the case of HSE-HAZOP, the preparation phase ends with obtaining this list, because the list indicates the HSE engineering action for each targeted item.

### 4.2. Risk-Analysis Phase

In this step, the possible events for each item and the expected consequences of risk exposure are determined.

#### 4.2.1. Category Classification of Items

The items constituting a process plant are divided into stationary and rotating machines according to their physical characteristics [24]. Additionally, from the perspective of HSE engineering, items can also be classified as a specific item accompanied by manual operation. Items that involve manual operation are more likely to be distinguished from each other, because the exposure of the operator to the risk occurs more frequently. The heater has a device machine characteristic; however, it has a high risk, as it contains a heating source. According to this criterion, Table 6 and Table 7 can be used.

In this step, HSE-HAZOP is used to determine the type of item to be analyzed.

#### 4.2.2. Possible Event Classification Based on Item Category

The analysis of a possible event of an HSE engineering issue is performed according to the classification of the item. There is process concerning, which is the cause of the mechanical limit of the item, and operation concerning, which is caused by the operation of the operator. Table 8 presents the HSE engineering issues based on the standardized item types in Table 6 and Table 7.

Table 8 presents only events that can adversely affect the human body, among the possible events for each item. This analysis is a modification of the classification of deviations based on the nodes in HAZOP.

#### 4.2.3. Selection of Possible Event

The human factors related to a process plant can be roughly classified into workers in the process plant and residents near the process plant. The workers in the process plant can be divided into those working in the open process area and those working inside the building. In the proposed HSE-HAZOP method, the subject of the possible event according to the characteristics of the item handling a lethal service is divided into three groups and analyzed.

#### 4.2.4. Derivation of Consequences

For items 1–3, experts from each discipline gather to derive the consequences. This procedure is borrowed from the HAZOP workshop. Table 9 presents a comparison between HAZOP and HSE-HAZOP.

### 4.3. Risk-Assessment Phase

In HSE-HAZOP, the method of risk assessment for the SIL is applied to the method of interpersonal damage. Four variables are considered for the personal injury of the SIL, and details are presented in Table 10.

Table 10 presents the risk parameters for SIL classification for the SIF; however, there is no semantic conflict in using it for risk analysis of human injury in HSE engineering. Therefore, this study borrows the descriptions of these parameters for SIL classification in HSE engineering.

**Figure 3:**
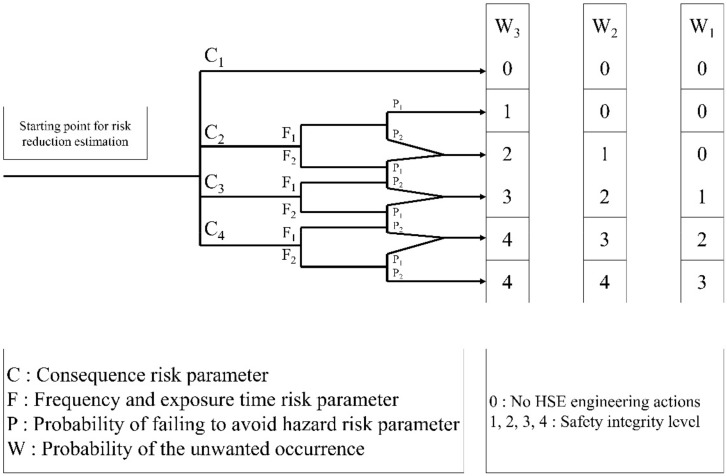
HSE-HAZOP: SIL Risk Graph for Personnel Safety.

Nevertheless, the SIL classification for the SIF presented in Figure 2 is contradictory to the application to HSE engineering. Hence, we modified it; specifically, SIL and SIL—were unified as SIL 0. In HSE engineering, it is not necessary to distinguish between SIL and SIL—, as for the SIF, because there is no need to have an actual application according to the risk. SIL 0 is applied to a safe situation, where no HSE engineering action is necessary. In the case of SIL b, SIL 4 should be modified and applied. This indicates that the SIF is insufficient and is not related to HSE engineering. Therefore, it is integrated with the grade of SIL 4 applied to the worst case in HSE engineering. The SIL risk graph for personnel safety that reflects this modification is shown in Figure 3.

In the HSE-HAZOP method, the final SIL rating is calculated using Table 10 and Figure 3 for the risk consequence of each item derived from 4.2.

### 4.4. Risk-Reduction Phase

#### 4.4.1. Category of HSE Engineering

The OSHA control of the exposure classification is transformed into the category of HSE engineering elements. It is practically impossible to apply the modified model to risk control level 4 of the OSHA, as the detailed design of the process plant is based on the design drawings of the licensor. Therefore, risk simulation using QRA and OCA is employed instead of selecting the major accident hazard to prevent further damage. Details are presented in Table 11.

**Figure 4:**
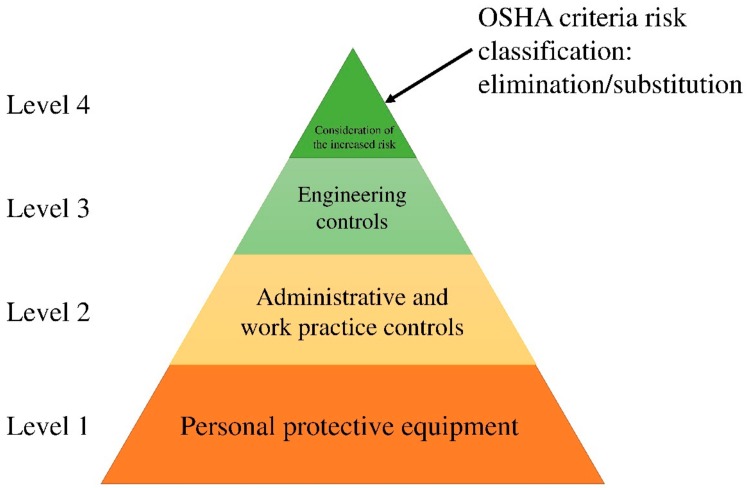
HSE-HAZOP criteria for risk classification.

Figure 4 compares the risk classification levels of the OSHA and HSE-HAZOP based on this modification.

Table 12 shows the detailed configuration of items for each HSE engineering level.

#### 4.4.2. Meaning of Risk Reduction

In this study, the level of HSE engineering described above was applied as one independent protection layer, which reduced the calculated SIL level. In HSE-HAZOP, HSE engineering is used to assess the SIL level indicated by the risk analysis, and ultimately, all risks are rendered to SIL 0. For example, if the risk-analysis result of SIL 3 is issued, a third step of risk reduction is needed to obtain SIL 0. In this case, a safety fence and sign, dike, toxic-gas detector, or damper inside the building should be applied in category 3 of Table 12, in consideration of the subject of exposure. As with HAZOP, in HSE-HAZOP, the HSE engineering technique is selected by the engineers of each discipline, through brainstorming.

However, in this study, the SIL level of the calculated risk was limited to take HSE engineering measures corresponding to one level. For example, a risk of SIL 3 indicates that the application of level 1 and 2 HSE engineering simultaneously to make SIL 0 is unsuitable. In HAZOP, the principle of double jeopardy is applied, i.e., multiple failures are not considered simultaneously. Applying this as a contrapositive from the viewpoint of HSE engineering means that it is impossible to use several levels of HSE engineering as a safeguard in terms of risk reduction for a single consequence. For example, if the result of risk assessment is SIL 4, it means that it is impossible to make SIL 0 by applying HSE engineering corresponding to level 1 and level 3 at the same time. The reason is that HSE engineering for level 1 and level 3 must be fully operational to reduce risk, and from a safety aspect, the fact that these two safeguards worked without problems is an incomplete assumption.

In addition, there is a prerequisite for level 4 HSE engineering to convert the SIL 4 risk into SIL 0. Level 4 HSE engineering consists of QRA and OCA. This is a simulation that cannot yield any risk reduction by itself. Separate studies on QRA and OCA may lead to recommendations for improvement that require process modifications. All these recommendations should be fulfilled to ensure SIL 0.

## 5. HSE-HAZOP Case Study

One part of a solution styrene butadiene rubber (SSBR) plant was used for a case study. The solvent in this part was used to increase the purity of the polymer in the process plant producing SSBR. HSE-HAZOP was applied to the process of removing and recycling this solvent.

### 5.1. HSE-HAZOP Preparation Phase

#### 5.1.1. Definition of Toxic Service

Information on the substances employed by the SSBR plant is presented in Table 13 and was used to determine whether they correspond to toxic category 3 or higher of UN GHS and ILO.

Heptanoic acid is the only chemical corresponding to category 3 or higher, according to the information in Table 3. Therefore, for this process, a stream that deals with heptanoic acid was selected from the HMB.

#### 5.1.2. Selection of Lethal Service through HMB

Table 14 presents the HMB values of the solvent removal and recycling process of the SSBR plant. Among the streams, only 3 and 5 deal with heptanoic acid, indicating that they involve a lethal service.

#### 5.1.3. Markup of Lethal Stream in PFD

**Figure 5:**
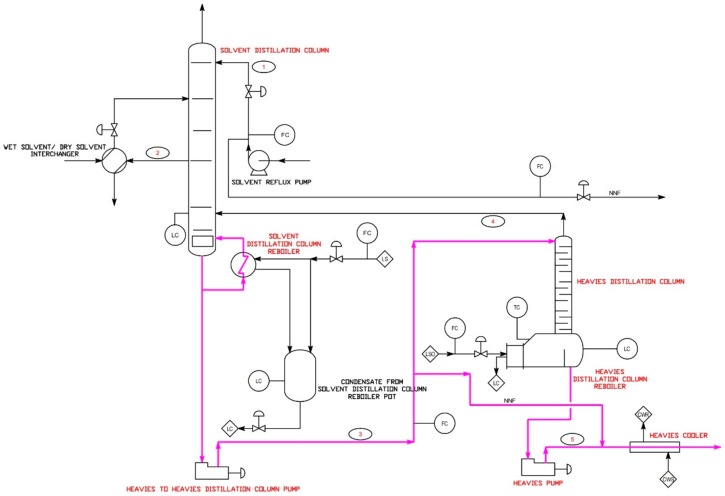
SSBR plant markup in PFD example.

Figure 5 shows the flow of the lethal-service markup in the PFD for solvent removal. The circulation processes in the SSBR plant are indicated and were used in this case study.

#### 5.1.4. Listing of Lethal Service Items

In the PFD of the SSBR plant, one must select an item that goes through the line corresponding to the lethal service. Because the proposed HSE-HAZOP method is conducted item-by-item, the items should be listed, and risk analyses should be performed sequentially. These items are “Solvent Distillation Column”, “Solvent Distillation Column Reboiler”, “Heavies to Heavies Distillation Column Pump”, “Heavies Distillation Column Reboiler”, “Heavies Distillation Column”, “Heavies Pump”, and “Heavies Cooler”.

### 5.2. Risk-Analysis Phase

In this case study, among the items involving a lethal service, we analyzed one item of the stationary-machinery type and one item of the rotating-machinery type using the HSE-HAZOP method. Solvent Distillation Column was selected as the stationary-machinery-type item and Heavies Pump was selected as the rotating-machinery-type item.

#### 5.2.1. Classification of Items

Solvent Distillation Column and Heavies Pump are not items corresponding to Table 6; thus, these items are not handled manually. These items are listed in Table 7. Solvent Distillation Column is the tower column (stationary machinery), and Heavies Pump is the rotating machinery pump.

#### 5.2.2. Classification of Possible Events and Exposure Group According to Specific Item Category

Table 8 presents possible events and targets to be considered in the HSE engineering according to the characteristics of the item. Table 8 applies to Solvent Distillation Column and Heavies Pump, which are examples considered in this case study.

#### 5.2.3. Derivation of Consequences

The brainstorming technique was used to derive consequences of possible events for the target exposure group identified by the engineers from different discipline gathered in the same way as the HAZOP workshop. Even if this is a possible event due to the same item, actual risk may not be generated, depending on the existence of an exposure group. Table 15 presents the results.

### 5.3. Risk-Assessment Phase

The risk was evaluated using the SIL technique for each consequence derived in Section 5.2. The final SIL level was selected by applying the method shown in Figure 3. In this case study, we applied the risk rating for the risk consequence of “operator in process area” due to the mechanical leak of Solvent Distillation Column as an example for “Heptanoic acid is a skin irritant and is dangerous upon exposure”.

#### 5.3.1. Consequence Risk Parameter

The nature of the column can cause a large number of serious injuries and deaths owing to the handling of large quantities of Heptanoic acid. C2 was selected.

#### 5.3.2. Frequency and Exposure Time Risk Parameter

It is unlikely that exposure to the column will occur, because the column and flanges are installed to prevent exposure. Therefore, F1 was chosen.

#### 5.3.3. Probability of Failing to Avoid Hazard Risk Parameter

In the case of exposure to heptanoic acid in the column, nearby operators may identify the risk and avoid it. Therefore, P1 was selected.

#### 5.3.4. Probability of the Unwanted Occurrence

It has been reported that more than one case of column exposure occurred in this process and a nearby process. Therefore, W3 was chosen.

**Figure 6:**
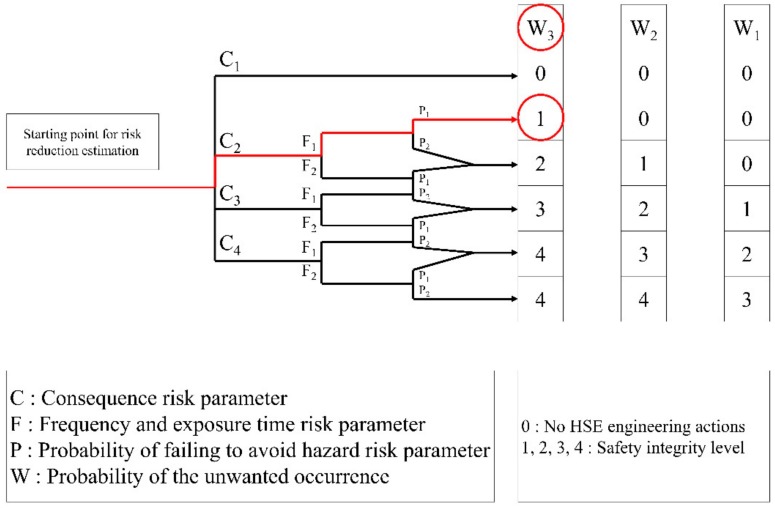
SSBR plant: SIL classification flowchart example.

The final SIL level was calculated as SIL 1 according to the aforementioned parameters (Figure 6). Table 15 presents the results of the risk analysis for the other consequences. In case of SIL 0, there is no problem without taking HSE engineering. However, if the calculation obtains SIL 1 and SIL 4, they should be engineered into SIL 0 to obtain a safe state via risk reduction.

### 5.4. Risk-Reduction Phase

SIL 1 and SIL 4 levels for each of the consequences analyzed in Table 15 in Section 5.2 should be engineered into SIL 0 through risk reduction. In the case of SIL 1, the item corresponding to HSE engineering level 1 should be applied. For SIL 4, the item corresponding to HSE engineering level 4 should be applied to reduce the risk to SIL 0. The measures for each HSE engineering level are presented in Table 12. The details regarding what action should be taken are to be selected according to the opinions of the engineers from different disciplines, as in the HAZOP workshop. In this example, it was decided to install an SSEW and PPE in the vicinity of the column for SIL 1 and perform OCA for SIL 4 to complement the process design. Details are presented in Table 15. OCA is scheduled to act on HSE engineering level 4 for SIL 4. One can only apply SIL 0 if action is taken according to the OCA for Solvent Distillation Column, and if the recommendations are outlined.

## 6. Conclusions

HSE engineering is required for maintaining human safety against risks arising from the operation of processing plants. However, each design product in HSE engineering is designed according to a simple engineering guide and local regulations, without a comprehensive analysis of the human risk. In this study, HSE-HAZOP, i.e., a methodology that can be applied to HSE engineering and employs the HAZOP and SIL techniques, was developed and applied to existing risk-analysis methods. The potential causes of human risks that may occur in a process plant were systematically analyzed using item categories, expected events, and exposure groups. The proposed method involves performing HSE engineering corresponding to the calculated SIL level. It has the advantage of being able to effectively prevent human risks. Additionally, the HSE-HAZOP worksheet integrates various design fields that constitute HSE engineering. The facile use of the safety management system of the employer or licensor is advantageous. The project management consultant and insurance companies in the process plant can review the HSE engineering application at a glance, according to the HSE-HAZOP worksheet, which can improve the efficiency of the design review. The most significant advantage of the proposed method is the prevention of personal injury related to the process plant. At present, there is no research paper on systematic HSE engineering in process plant design. Therefore, in this paper, if a systematic study technique considering the safety of the operator is proposed in the design of the process plant and it is developed though the follow-up studies, it may contribute to the safety of the process plant.

## Figures and Tables

**Table 1:** Major disciplines of engineering in the process-plant industry.

Discipline	Activity
Process	Overall process system design
Equipment/mechanical	Mechanical item design in the process plant
HSE	Technical safety design related to the process plant
	Fire safety design related to the process plant
Civil	Civil factors related to the design of the process plant
Piping	Plant layout, plant piping layout, 3D piping modeling, piping material
Instrument and control	Instrument and control design of the process plant
Electrical	Electrical design of the process plant

**Table 2:** HSE engineering parts.

No.	HSE Engineering Parts
1	Safety shower and eyewash (SSEW)
2	Personal protective equipment (PPE)
3	Self-contained breathing apparatus (SCBA)
4	Evacuation, escape, and rescue analysis (EERA)
5	Workplace monitoring system
6	Safety fence and sign
8	Dike
9	Toxic-gas detector
10	Quantitative risk assessment (QRA) for toxic-gas dispersion
11	Offsite consequence analysis (OCA)

**Table 3:** Toxic service classification criteria according to the specifications of Shell.

If Swallowed	If in Contact with Skin	If Inhaled
LD50 oral, rat ≤5 mg/kg	LD50 dermal, rat or rabbit ≤50 mg/kg	LC50 inhalation, rat, for gases ≤100 ppm·mol/4 h
		LC50 inhalation, rat, for vapors ≤0.5 mg/L/4 h
		LC50 inhalation, rat for particulates or aerosols ≤0.05 mg/L/4 h

**Table 4:** Acute toxicity hazard categories and approximate LD50/LC50 values defining the respective categories.

Exposure Route	Category 1	Category 2	Category 3	Category 4	Category 5
Oral (mg/kg)	5	50	300	2000	5000
Dermal (mg/kg)	50	200	1000	2000	N/A
Gas (ppm)	100	500	2500	5000	
Vapor (mg/L)	0.5	2	10	20	
Dust and mist (mg/L)	0.05	0.5	1	5	

**Table 5:** Additional information for the categories.

Description	Category 1	Category 2	Category 3	Category 4	Category 5
Symbol	Skull and crossbones	Skull and crossbones	Skull and crossbones	Exclamation mark	No symbol is used
Signal word	Danger	Danger	Danger	Warning	Warning
Hazard statement: -Oral	Fatal if swallowed	Fatal if swallowed	Toxic if swallowed	Harmful if swallowed	May be harmful if swallowed
-Dermal	Fatal in contact with skin	Fatal in contact with skin	Toxic in contact with skin	Harmful in contact with skin	May be harmful in contact with skin
-Inhalation	Fatal if inhaled	Fatal if inhaled	Toxic if inhaled	Harmful if inhaled	May be harmful if inhaled

**Table 6:** Item list for manual operation.

Item Type	Description for Manual Handling
Chemical injection package	Chemical injection involves manual handling of the connection of the injection point and the connection to the chemical tank.
Filter	Manual handling comprises the periodic replacement of filters.
Reactor	If the reactor operates with a catalyst, the operator must perform manual handling by periodically replacing the catalyst.
Tank loading/unloading	Injecting material from the tank or entering the tank involves a hose reel, which requires manual handling where workers may be exposed to the chemical.

**Table 7:** Item classification for the standardized process plant.

Main Category	Description
Rotating machinery	Pump
	Compressor
Stationary machinery	Drum
	Vessel
	Storage tank
	Tower/column
	Heat exchanger
	Air cooler
Heater	Electrical heater
	Fired heater

**Table 8:** Expected risk cause and consequence by item type in HSE Engineering.

Main Category	Item Type	Possible Event		
		Cause Type	Description	
Manual handling item	Chemical injection package	Process-related	1	Mechanical leak
		Operation-related	1	Human error
	Filter	Process-related	1	Mechanical leak
			2	Asphyxiation
	Reactor (concerned with catalyst replacement)	Process-related	1	Mechanical leak
			2	Thermal/cryogenic effect
			3	Asphyxiation
	Tank loading/unloading (concerned by manual hose connection)	Process-related	1	Mechanical leak
		Operation-related	1	Human error
Rotating machinery	Pump	Process-related	1	Mechanical leak
			2	Thermal/cryogenic effect
		Operation-related	1	Human error
	Compressor	Process-related	1	Mechanical leak
			2	Thermal/cryogenic effect
			3	Asphyxiation
		Operation-related	1	Human error
Stationary machinery	Drum	Process-related	1	Mechanical leak
	Vessel			
	Storage tank			
	Tower/column			
	Heat exchanger		2	Thermal/cryogenic effect
	Air cooler		3	Asphyxiation
Heater	Electrical heater	Process-related	1	Thermal/cryogenic effect
	Fired heater			

**Table 9:** Comparison between HAZOP and HSE-HAZOP.

HAZOP	HSE-HAZOP	Comparison
Node	Lethal service	An HAZOP node is classified according to process issues; however, HSE-HAZOP regards the toxic service based on specific criteria as a node. In other words, HSE-HAZOP marks the stream that handles toxic service on the PFD and selects it as the base for analysis of accident scenarios.
Item	Item category	HAZOP does not define the cause of the risk according to the characteristics of the item across nodes; however, HSE-HAZOP classifies HSE engineering issues according to the item category. In other words, HSE-HAZOP performs a risk analysis considering the characteristics of item category across the stream handling toxic services on the PFD.
Deviation	Possible event	HAZOP involves analysis based on the deviations, regardless of the characteristics of the items; however, HSE-HAZOP classifies the possible events according to the characteristics of the item. For example, in the case of a tank loading/unloading facility, manual operation of the operator is involved, so human error is considered, but if the operator’s direct operation is not involved in process operation such as a heater, human error is not considered.
-	Exposure target	HAZOP does not classify the risk object separately; however, HSE-HAZOP classifies the risk object into three categories. (Operator in the process area, Operator inside the building, Local resident)
Consequences		In both HAZOP and HSE-HAZOP, the consequences based on the risk are derived by engineers from each discipline, via brainstorming.

**Table 10:** Risk parameters in the SIL classification.

Risk Parameter		Classification
Consequence (C)	C1	Light injury to persons
	C2	Serious permanent injury to one or more persons; death of one person
	C3	Death of several persons
	C4	Catastrophic effect; death of many people
Probability of avoiding the hazardous event (P)	P1	Avoidable
	P2	Unavoidable
Frequency of presence in the hazardous zone multiplied by the exposure time (F)	F1	Rare-to-more frequent exposure in the hazardous zone
	F2	Frequent-to-permanent exposure in the hazardous zone
Probability of the unwanted occurrence (W)	W1	Demand rate of <0.1 per year
	W2	Demand rate between 0.1 and 1 per year
	W3	Demand rate between 1 and 10 per year

**Table 11:** Comparison between the OSHA criteria risk classification and HSE-HAZOP criteria risk classification.

OSHA Criteria Risk Classification			HSE-HAZOP Criteria Risk Classification	
Degree	Title	Description	Title	HSE Engineering Description
Level 1	PPE	Use protection to reduce exposure to risk factors	PPE	Comprehensive PPE designed to reduce risk directly when workers are exposed to risk
				The SSEW, PPE, SCBA can be applied.
Level 2	Administrative and work practice controls	Establish efficient processes or procedures	Administrative and work practice controls	A plan to reduce risk via the training and management of workers
				EERA can be improved from a safety-management standpoint as a measure to ensure safety in response to evacuation and emergency situations of workers.
				It can be used for work schedule and job assignment of workers by utilizing it for workplace monitoring. This is the same intent as for OSHA Level 2.
Level 3	Engineering controls	Implement physical change to the workplace, which eliminates/reduces the hazard of the job/task	Engineering controls	This is to ensure the safety of workers through improvement of the process design. This is a higher level of protection than direct operator protection, such as Level 1 actions that take direct action on workers.
				Owing to the installation of structures such as the safety fence and sign, workers are not exposed to risk.
				By installing a dike, the safety of workers can be ensured by limiting the range of exposure.
				By installing the toxic-gas detector, it is possible to reduce the risk by providing information regarding the safety situation directly to the workers or by linking with the process system (emergency shut down, etc.).
Level 4	Elimination/substitution	Substitute with safer alternatives	Consideration of the increased risk	It is difficult to replace the chemicals handled in the process plant with other substances in HSE engineering, because the design of the licensor is used to produce desired products through the process reaction of each substance.
				We propose QRA and OCA as HSE engineering measures to consider the situations that can spread risk from the risk source and assign a risk to the environment according to the concept of risk expansion. On the basis of the simulation results obtained via the QRA and OCA, detailed action items must be discussed.

**Table 12:** HSE engineering actions according to the OSHA modified control of exposure.

Risk Level	HSE Engineering Action According to the Risk Level
Level 1	SSEW
	PPE
	SCBA
Level 2	Operating manual
	EERA
	Workplace monitoring system
Level 3	Safety fence and sign
	Dike
	Toxic-gas detector
	Damper inside the building
Level 4	QRA for toxic-gas dispersion
	OCA

**Table 13:** Chemicals in SSBR solvent-related process.

No.	Chemical	CAS No.	LD50 (oral) [mg/kg]	LD50 (dermal) [mg/kg]	LC50 Gases (ppm) Vapors (mg/L)
1	13-Butadiene	106-99-0	5480	N/A	129,000 ppm
2	Cyclopentane	287-92-3	11,400	N/A	N/A
3	Styrene	100-42-5	2650	5010	12 (mg/L)
4	THFee	62435-71-6	N/A	N/A	N/A
6	Heptanoic acid	111-14-8	N/A	N/A	>4.6 (mg/L)

**Table 14:** SSBR plant HMB example.

Stream No.	1	2	3	4	5
Component	kg/hr	kg/hr	kg/hr	kg/hr	kg/hr
13-Butadiene	630.9	0.0	0.0	0.0	0.0
Cyclopentane	5332.1	22,005.7	442.7	427.5	15.2
Styrene	0.0	0.1	1.9	0.1	1.8
THFee	0.0	0.1	8.4	0.2	8.3
Heptanoic acid	0.0	0.0	0.3	0.0	0.3
Total	5963.0	22,005.9	453.3	427.8	25.6

**Table 15:** SSBR Plant HSE-HAZOP Worksheet Example.

Main Category	Item Type	Possible Event			Exposure Group			Consequence	Risk Consideration					Risk Reduction		
		Cause Type	Description						Consequence Risk Parameter	Frequency and Exposure Time Risk Parameter	Probability of Failing to Avoid Hazard Risk Parameter	Probability of the Unwanted Occurrence	Final SIL Level	HSE Engineering Level	Detail Action	Final Risk Level
Solvent Distillation Column Stationary machinery	Tower/ column	Process-related	1	Mechanical leak	Inside the plant	1	Operator in the process area	Heptanoic acid is a skin irritant and is dangerous upon exposure.	C2	F1	P1	W3	SIL 1	Level 1	Install SSEW near the column	SIL 0
						2	Operator inside the building	No effect (There is no occupied building near this item.)								
					Outside the plant	1	Local resident	Because there is a large amount of heptanoic acid in the column, an additional study on external damage is needed.	C4	F1	P2	W3	SIL 4	Level 4	OCA is required	SIL 0
			2	Thermal/cryogenic effect	Inside the plant	1	Operator in the process area	No effect (There is no direct exposure to the operator, owing to the structure of the column.)								
						2	Operator inside the building	No effect (There is no occupied building near this item.)								
					Outside the plant	1	Local resident	No Effect (There is no need to consider the thermal/cryogenic effect on the local community near this item.)								
			3	Asphyxiation	Inside the plant	1	Operator in the process area	Damage due to the operator choking has been reported during the maintenance work in the column.	C2	F1	P1	W3	SIL 1	Level 1	Install PPE near the column	SIL 0
						2	Operator inside the building	No effect (There is no occupied building near this item.)								
Heavies Pump Rotating machinery	Pump	Process-related	1	Mechanical leak	Inside the plant	1	Operator in the process area	Heptanoic acid is a skin irritant and is dangerous upon exposure.	C1	F2	P1	W2	SIL 0	No action required	No action required	SIL 0
						2	Operator inside the building	No effect (There is no occupied building near this item.)								
					Outside the plant	1	Local resident	No effect (The volume of fluid is insufficient for performing risk analysis.)								
			2	Thermal/cryogenic effect	Inside the plant	1	Operator in the process area	Skin burned owing to the surface temperature of this item.	C1	F1	P1	W2	SIL 0	No action required	No action required	SIL 0
						2	Operator inside the building	No effect (There is no occupied building near this item.)								
					Outside the plant	1	Local resident	No effect (The volume of fluid is insufficient for performing risk analysis.)								
		Operation-related	1	Human error	Inside the plant	1	Operator in the process area	Physical harm to the operator due to the high pressure of this item.	C1	F2	P1	W2	SIL 0	No action required	No action required	SIL 0
						2	Operator inside the building	No effect (There is no occupied building near this item.)								
